# Anticancer activity of an extract from needles and twigs of *Taxus cuspidata *and its synergistic effect as a cocktail with 5-fluorouracil

**DOI:** 10.1186/1472-6882-11-123

**Published:** 2011-12-02

**Authors:** Weihu Shang, Jinping Qiao, Chenxin Gu, Wei Yin, Jinglei Du, Wei Wang, Meilin Zhu, Mei Han, Weidong Lu

**Affiliations:** 1 Key Laboratory of Radiopharmaceuticals, Ministry of Education, College of Chemistry, Beijing Normal University, Beijing 100875, China; 2 Leonard P. Zakim Center, Dana-Farber Cancer Institute, Harvard Medical School, Boston, MA 02115, USA

## Abstract

**Background:**

Botanical medicines are increasingly combined with chemotherapeutics as anticancer drug cocktails. This study aimed to assess the chemotherapeutic potential of an extract of *Taxus cuspidata *(*TC*) needles and twigs produced by artificial cuttage and its co-effects as a cocktail with 5-fluorouracil (5-FU).

**Methods:**

Components of *TC *extract were identified by HPLC fingerprinting. Cytotoxicity analysis was performed by MTT assay or ATP assay. Apoptosis studies were analyzed by H & E, PI, TUNEL staining, as well as Annexin V/PI assay. Cell cycle analysis was performed by flow cytometry. 5-FU concentrations in rat plasma were determined by HPLC and the pharmacokinetic parameters were estimated using 3p87 software. Synergistic efficacy was subjected to median effect analysis with the mutually nonexclusive model using Calcusyn1 software. The significance of differences between values was estimated by using a one-way ANOVA.

**Results:**

*TC *extract reached inhibition rates of 70-90% in different human cancer cell lines (HL-60, BGC-823, KB, Bel-7402, and HeLa) but only 5-7% in normal mouse T/B lymphocytes, demonstrating the broad-spectrum anticancer activity and low toxicity to normal cells of *TC *extract *in vitro*. *TC *extract inhibited cancer cell growth by inducing apoptosis and G_2_/M cell cycle arrest. Most interestingly, *TC *extract and 5-FU, combined as a cocktail, synergistically inhibited the growth of cancer cells *in vitro*, with Combination Index values (CI) ranging from 0.90 to 0.26 at different effect levels from IC50 to IC90 in MCF-7 cells, CI ranging from 0.93 to 0.13 for IC40 to IC90 in PC-3M-1E8 cells, and CI < 1 in A549 cells. In addition, the cocktail had lower cytotoxicity in normal human cell (HEL) than 5-FU used alone. Furthermore, *TC *extract did not affect the pharmacokinetics of 5-FU in rats.

**Conclusions:**

The combinational use of the *TC *extract with 5-FU displays strong cytotoxic synergy in cancer cells and low cytotoxicity in normal cells. These findings suggest that this cocktail may have a potential role in cancer treatment.

## Background

Cancer is a multifactorial disease that requires a multi-targeted therapeutic approach [1,2]. Chemotherapy has undergone a gradual transition from mono-substance therapy toward multidrug therapy, and drug cocktails strategy has become widely adopted. Properly formulated drug combinations are believed to enhance synergy, and interactions of chemical components within the combination may improve therapeutic efficacy over single drugs [3-6]. Botanical medicines are generally plentiful, low cost, and relatively non-toxic in clinical practice, and in many cases plant extracts are thought to be therapeutically superior to their single isolated constituents [7,8]. Therefore, botanical medicines are increasingly combined with chemical medicines in anticancer drug cocktails, especially in countries where botanical medicines are well-accepted [9,10]. Some studies have suggested that for cancer treatment, drug cocktails combining botanical and chemical medicines may exhibit enhanced efficacies with diminished side effects and complications [11-13].

*Taxus cuspidata (TC)*, also called Japanese yew, is an evergreen tree with anticancer and anti-inflammatory activities [14-16]. While *TC *is scarce as a natural resource, artificial cuttage is reproducible and makes *TC *needles and twigs constantly available. DaKeSu, a *TC *extract of *TC *needles and twigs produced by artificial cuttage, has been under preclinical and clinical investigation in China as a botanical medicinal product [17,18]. Chinese language sources have reported animal-based and preclinical studies showing DaKeSu activity against breast, lung, and digestive tract cancers [17,18], but the anticancer spectrum and mechanism of the extract have not been studied in detail.

5-Fluorouracil (5-FU) is one of the most commonly used drugs for treatment of breast, digestive tract, and other cancers [19-21]. It is often used clinically in combination with other agents such as paclitaxel, docetaxel, and cisplatin [22-24]. A few studies have shown synergistic effects of combinations of 5-FU with botanical medicines or components thereof. For example, oroxylin A, a bioactive *Scutellaria baicalensis *Georgi flavonoid, has synergistic effect with 5-FU on HepG2 human hepatocellular carcinoma and on H_22 _transplanted mice [25]. Chan-Yu-Bao-Yuan-Tang, an herbal medicine formula, induced apoptosis synergistically with 5-FU in lung and cervical cancer cells [26]. Though botanical medicines and 5-FU are both commonly used in clinical practice, there have been far fewer studies combining 5-FU and botanical medicines than on 5-FU or botanical medicines alone.

The aim of this paper is to evaluate the efficacy of the extract of *TC *needles and twigs produced by artificial cuttage as a source of useful anticancer agents and the co-efficacy at the cellular level of a cocktail combining *TC *extract and 5-FU. We also assessed whether *TC *extract would influence the pharmacokinetics of 5-FU in animals. These results show the utility for identifying herb-chemotherapeutic drug combinations.

## Methods

### Reagents, cell lines, and animals

5-FU (99.9% purity) was purchased from Shanghai Bangcheng Chemical Co., Ltd. (Shanghai, China). The *TC *extract was kindly provided by China Hongdoushan Tech. Co., Ltd. (Heilongjiang, China). HPLC-grade methanol and acetonitrile were purchased from Fisher Scientfic (Fair Lawn, NJ, USA).

The human cancer cell lines, MCF-7 (breast), PG and A549 (lung), PC-3M-1E8 (prostate), BGC-823 (gastric), WM451 (melanoma), Bel-7402 (hepatocellular), KB (oral squamous), HeLa (cervical), and HL-60 (leukemic), and the normal cells, mouse spleen T lymphocytes (T cells), mouse spleen B lymphocytes (B cells), and a human embryonic lung cell line (HEL), were kindly provided by the Department of Pathology, Peking University Health Science Center. The cells were maintained in RPMI-1640 medium supplemented with 10% fetal bovine serum (Gibco, Carlsbad, CA), 100 IU/ml penicillin and 100 IU/ml streptomycin in 5% CO_2 _humidified atmosphere at 37°C.

Ten male Sprague-Dawley rats (6-7 weeks old, 200-250 g) were purchased from Vital River (Beijing, China). Five rats in the control group received physiological saline orally (20 ml/kg b.i.d. for 8 days), and the other 5 rats in the *TC *extract-pretreated group received a suspension of *TC *extract orally (0.25 g/kg b.i.d. for 8 days). After the 8-day treatment period, both groups of rats received 5-FU (48 mg/kg) by intraperitoneal (i.p.) injection after a 12-h fast. All animals were maintained according to the international guidelines for care and use of laboratory animals and all experimental procedures involving animals were approved by the Ethics Committee of Beijing Normal University (BNU/EC/01/2011).

### Extract identification

For HPLC fingerprinting, *TC *extract was dissolved in methanol and filtered through membrane filters (0.45 μm pore size). A Waters HPLC system equipped with a 1525 pump and a 2487 UV detector (Waters, USA) was used. A C18 column (4.6 mm × 250 mm, 5 μm, Kromasil) was used as the solid phase, the mobile phase consisted of CH_3_CN-water (50:50, v/v) (A) and CH_3_CN-water (15:85, v/v) (B), and the elution gradient profile was as follows: 0-15 min, 0% A; 15-20 min, 10% A; 20-40 min, 25% A; 40-60 min, 40% A; 60-80 min, 50% A; 80 min, 100% A. The flow rate was 0.8 ml/min, the column temperature was room temperature, the injection volume was kept at 10 μl; the scan wavelength was set from 190 to 370 nm, and the detection wavelength was set at 227 nm.

### Cell viability assay

The cell viability was evaluated for both cancer and normal cell lines by MTT assay on 96-well plates [27] or ATP assay on 384-well plates as previously described [28]. For the MTT assay, cells were seeded on 96-well plates at 4 × 10^4 ^cells/ml over night. Then cells were treated with 10 μg/ml *TC *extract for 72 h. 10 μl of a 5 mg/ml MTT solution was added to each well and the plate was incubated at 37°C for 4 h. Subsequently, 100 μl 0.1% NH_4_Cl and 10% dodecyl phenyl sodium sulfonate solution was added to each well, and the plates were incubated overnight at 37°C. Absorbance was detected using a Victor^3 ^V Multilabel reader (PerkinElmer, USA) with the filter set to 570 nm (reference wavelength 650 nm). The ATP assay used the CellTiter-Glo^® ^Luminescent Cell Viability Assay kit (Madison, WI, USA). Briefly, cells were seeded on 384-well plates at 2 × 10^4 ^cells/ml over night. Various concentrations of single 5-FU, single *TC *extract or combination (1:1) in the same volume (0.1, 0.3, 1, 3, 10, 30,100, 300 μg/ml) were added and the plates were incubated at 37°C for 72 h. The detection protocol included the addition of 10 μl of the working solution of the ATP kit to each well. The luminescence of each well was measured using a Victor^3 ^V Multilabel reader (PerkinElmer, USA).

### Histology and immunohistochemistry

Cells were cultured in chamber slides on 6-well plates at 5 × 10^5 ^cells/ml and allowed to attach overnight, followed by treatment with 10 μg/ml *TC *extract for 24 h. Cells were washed with phosphate-buffered saline (PBS), fixed with freshly prepared ice-cold 4% paraformaldehyde, and then stained with H & E (St. Louis, MO, USA). Cells were observed using a Leica DM-RXA microscope.

Cells were treated with *TC *extract for 24 h, harvested by trypsinization, washed twice with PBS, and fixed for 1 h in 1% paraformaldehyde. After RNase treatment, the cells were adjusted to a density of 1 × 10^6 ^cells/ml, and then stained with PI (St. Louis, MO, USA). Immunofluorescent images were acquired using a Leica TCSNT confocal microscopy.

TUNEL staining was performed using an Apo-Direct kit (San Diego, USA) according to the manufacturer's instructions. Briefly, cells were cultured in chamber slides on 6-well plates at 5 × 10^5 ^cells/ml over night. Then cells were treated with 10 μg/ml *TC *extract for 24 h. The cells were fixed with 4% paraformaldehyde for 60 min at room temperature, washed 3 times with PBS, permeabilized with 0.1% Triton X-100 in 0.1% sodium citrate, and then rinsed with PBS. Cells were stained with 50 μl TUNEL reaction mixtures at 37°C for 60 min and washed with PBS. Afterwards, the cells were viewed using an optical microscope.

### Annexin V/PI Assay

Annexin V/PI assay was determined using the Annexin-V FITC Staining kit (Beijing Biosea Biotechnology Co., LTD, China) in combination with propidium iodide, according to the manufacturer's instructions. Briefly, cells were cultured in T25 flasks at a density of 5 × 10^5 ^cells/ml and allowed to attach overnight, followed by treatment with 10 μg/ml *TC *extract for 48 h. Cells were washed with PBS and incubated with Annexin-V FITC labeling solution (containing 10 μl Annexin-V- FITC labeling reagent in 300 μl incubation buffer) for 15 min at room temperature and then added 5 μl propidium iodide solution. Analysis was carried out by flow cytometry (FACS Calibur, Becton-Dickinson, USA) and confocal microscopy (Leica, Germany).

### Flow cytometry analysis

Cells were cultured in T25 flasks at a density of 5 × 10^6 ^cells/ml and treated with a series of different concentrations of *TC *extract (0, 1.67, 4.16, 8.33, 33.33, and 66.67 μg/ml) for 24 h. The cells were harvested by trypsinization and washed twice with PBS. For cell cycle and apoptosis assays, cells were fixed gently in cold 70% ethanol at 4°C overnight and then re-suspended in PBS with 0.1 mg/ml RNAse A and 0.1% Triton X-100 and incubated at 37°C for 30 min. The cell cycle phase and presence of apoptotic nuclei were determined by staining with PI. Flow cytometry analyses were performed using a FACSCalibur instrument and CellQuest software (Becton Dickinson, USA).

### Pharmacokinetic study

Blood samples (0.5 ml) were collected from the retro-orbital plexus of rats under light ether anaesthesia into heparinized tubes at 0, 1, 3, 5, 10, 20, 30, 60, 90, 120, and 240 min after dosing with 5-FU. Blood samples were centrifuged at 7000 rpm for 5 min. Drug-containing plasma (200 μl), 200 μl (1 mg/ml) internal standard (IS) solution, and 600 μl methanol were added to a 2-ml centrifuge tube for the assay. The sample was shaken for 1 min using a vortex mixer and then extracted with ethyl acetate/isopropanol (9:1, v/v). The mixture was then centrifuged at 3000 rpm for 5 min, and the upper layer was pipetted into a clean 5-ml centrifuge glass tube. The sample was then extracted twice more in the same way. The supernatant was mixed and evaporated to dryness under nitrogen at 40°C. The dried residues were reconstituted in 200 μl of methyl cyanide/methanol (75:25, v/v), vortex-mixed for 10 s, and centrifuged at 3000 rpm for 5 min, and the supernatant was analyzed by HPLC.

The plasma concentrations of 5-FU were determined by HPLC. The HPLC system comprised a 1525 pump and 2487 UV detector (Waters, USA). An Atlantis DC C18 column (3 μm, 30 mm × 2.1 mm, Waters, USA) was used. The mobile phase consisted of 10 mM ammonium acetate in water (A) and acetonitrile (B). The gradient program was linearly increased from 10% B to 80% B in the first 2 min, held for 2 min, and then returned to 10% B. The flow rate was 0.2 ml/min. The end time of the program was set at 6 min. and the detection wavelength was 254 nm [29].

### Combination index (CI) for determining synergism additivity or antagonism

The combined effects of *TC *extract and 5-FU were subjected to median effect analysis with the mutually nonexclusive model as previously described [30]. The combination index (CI) for determining synergism and antagonism between the substances was calculated using Calcusyn1 software (ver 1.1.1, 1996; Biosoft Inc., Cambridge, UK). CI < 1, CI = 1, and CI > 1 indicated synergism, additivity, and antagonism, respectively. The results by ATP assay were analyzed for CI determination.

### Statistical analysis

The significance of differences between values was estimated by using a one-way ANOVA. P < 0.05 was considered statistically significant.

## Results

### Component identification of *TC *extract by HPLC fingerprinting

**Figure 1:**
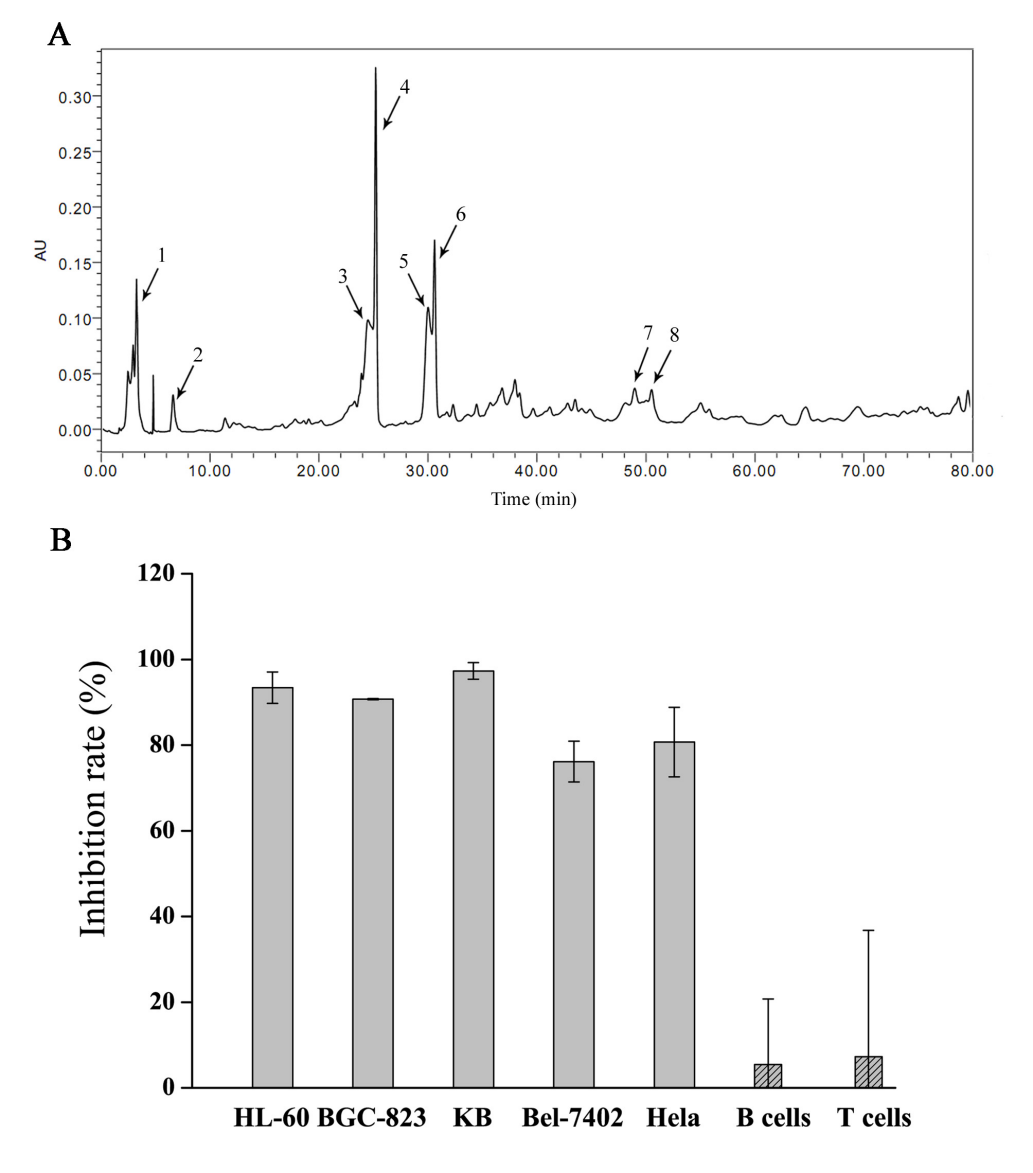
**Chemical composition and cytotoxic activity of *TC *extract**. (A) Peaks: 1, taxine A (5.00%); 2, 10-DAB III (1.45%); 3, 9-hydroxyl-13-acetylbaccatin III (9.38%); 4, 7-xyl-10-DAT (9.63%); 5, baccatin III (8.32%); 6, 10-DAT (6.31%); 7, cephalomannine (2.67%); 8, paclitaxel (2.25%). (B) Differences in *TC *extract cytotoxicity in 5 human cancer cell lines and normal mouse spleen B and T lymphocytes. The cell growth inhibition was quantified by the MTT assay. Cells were treated with 10 μg/ml *TC *extract for 72 h. The data shown are from 3 independent experiments.

Chromatographic fingerprinting is a powerful technology for authentication of natural products. The application of chromatographic fingerprinting in component identification of natural products continues to expand. HPLC fingerprinting of *TC *extract for quality control is shown in Figure 1A. The 8 main compounds of *TC *extract found in this study are in agreement with previous reports [9,31-33]. The taxoids contained high proportions of 7-xyl-10-DAT (9.63%), 9-hydroxyl-13-acetylbaccatin III (9.38%), and baccatin III (8.32%), moderate amounts of 10-DAT (6.31%) and taxine A (5.00%), and low amounts of cephalomannine (2.67%), paclitaxel (2.25%), and 10-DAB III (1.45%). The structures of these compounds are shown in Additional file 1.

### *TC *extract inhibits growth of human cancer cells but not normal cells

To explore the effects of *TC *extract on human cancer cell lines and normal cells *in vitro*, the cytotoxicity of *TC *extract at 10 μg/ml for 72 h was assessed by MTT assays in a panel of human cancer cell lines namely HL-60, BGC-823, Bel-7402, KB, HeLa and normal mouse T/B lymphocytes. Figure 1B shows that growth was strongly inhibited in all cancer cells by 93% for HL-60 cells, 90% for BGC-823 cells, 97% for KB cells, 76% for Bel-7402 cells, and 80% for HeLa cells. In contrast, growth of mouse spleen T/B lymphocytes was inhibited only 7% and 5%, respectively. Therefore, *TC *extract showed strong and broad-spectrum anticancer activity with low toxicity to normal cells.

### *TC *extract induces apoptosis and arrests human cancer cells at the G_2 _/M phase

**Figure 2:**
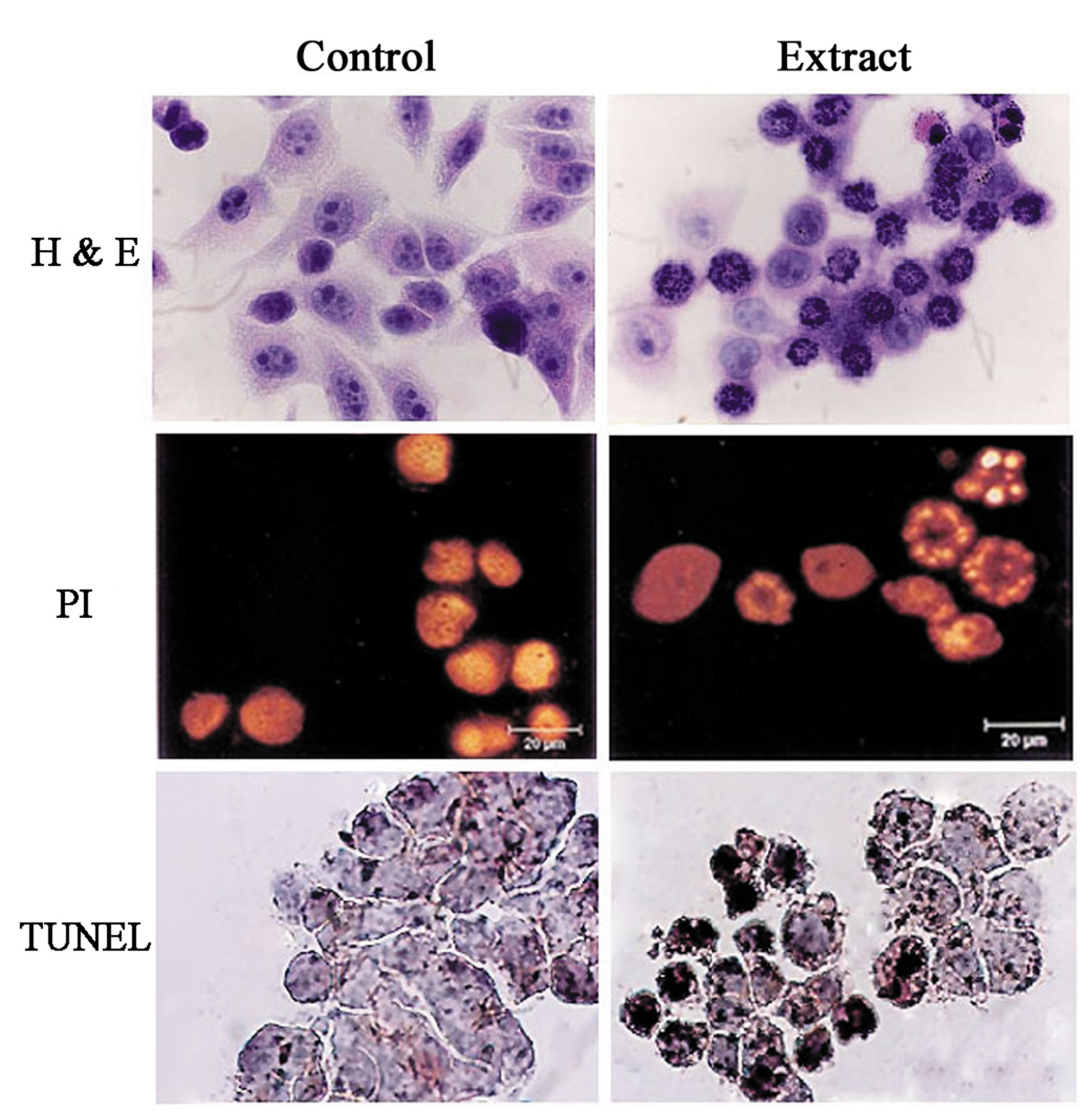
**Apoptosis detected by hematoxylin and eosin (H & E), propidium iodide (PI), and TUNEL staining**. PC-3M-1E8 cells were treated with 10 μg/ml *TC *extract for 24 h. Cells were processed for H & E staining (top panel), PI staining (middle panel) and TUNEL reaction (bottom panel). Apoptotic cells showed condensed and fragmented nuclei and positive TUNEL reaction. The H & E and TUNEL staining were observed by optical microscopy (magnification, × 200). The results of PI analysis were observed by a confocal microscopy. Scale line = 20 μm.

We next performed 3 separate apoptosis assays, H & E, PI, and TUNEL staining, to determine whether *TC *extract inhibited cell growth by inducing apoptosis. Treatment with 10 μg/ml of *TC *extract for 24 h produced significant morphological changes in PC-3M-1E8 cells compared with control cells. As shown in Figure 2, *TC *extract-treated cells displayed cellular shrinkage and nucleoli ambiguity by H & E staining, nuclear fragmentation by PI staining, and TUNEL-positive staining, all typical characteristics of apoptosis, compared with control cells. Similar effects were seen in PG and WM451 cells (data not shown). These results demonstrated that *TC *extract inhibited cell growth by inducing apoptosis.

**Figure 3:**
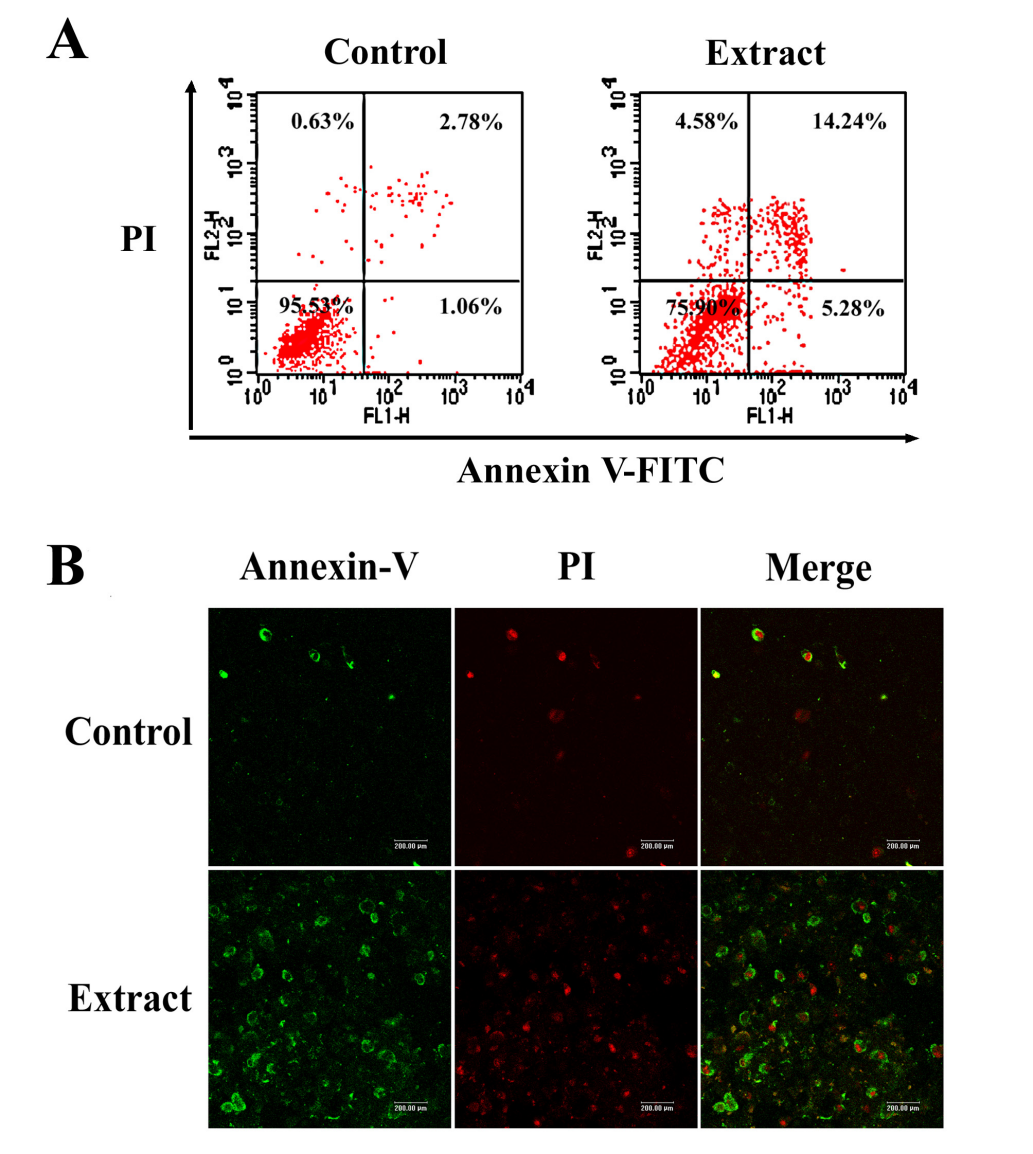
**Apoptotic percentage distribution and observation by Annexin V/PI assay**. PC-3M-1E8 cells were treated with 10 μg/ml *TC *extract for 48 h and stained with annexin V-FITC antibody (green staining) and propidium iodide (red staining) and analysed by flow cytometry (A) and observed using a confocal microscopy (B). Scale line = 200 μm.

To further measure percentage distribution of *TC *extract-induced apoptotic and necrotic human cancer cells, Annexin V/PI assay was used. Figure 3A shows specific percentage distributions of PC-3M-1E8 cells as obtained by flow cytometry. Moreover, confocal microscopy analysis (Figure 3B) demonstrated that significant apoptosis occured in *TC *extract-treated PC-3M-1E8 cells as depicted by strong reaction of these cells with the Annexin V antibody (green) compared to control cells. Some propidium iodide staining (red) was also noted in the cells, indicating very late stage apoptosis or necrosis.

**Figure 4:**
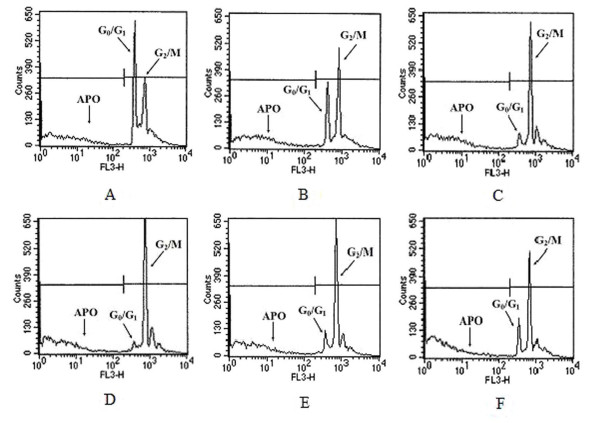
***TC *extract induced apoptosis in a dose-dependent manner and arrested the cell cycle at G_2_/M**. The sub-DNA apoptotic peak, G_0_/G_1 _peak, and G_2_/M peak were quantified by flow cytometry after treatment of PC-3M-1E8 cells for 24 h with the following concentrations of *TC *extract: 0 μg/ml as a control (A), 1.67 μg/ml (B), 4.16 μg/ml (C), 8.33 μg/ml (D), 33.33 μg/ml (E), and 66.67 μg/ml (F). The cells were stained with propidium iodide and analysed by flow cytometry.

We also performed sub-DNA and cell cycle analysis by flow cytometry in PC-3M-1E8 cells treated with different concentrations of *TC *extract for 24 h. As shown in Figure 4, the cell population in sub-G1 (APO in Figure 4) gradually increased with *TC *extract treatment in a dose-dependent manner, indicating increasing degrees of apoptosis triggered by the extract. As shown in Figure 4 and Table 1, we also observed gradual dose-dependent decreases in the G_0_/G_1 _phase population (23%-6%) as compared with a negative control (36%) and increases in the G_2_/M phase population (64%-84%) as compared with a negative control (50%) with increasing concentrations of *TC *extract, indicating that the cell cycle was arrested at the G_2_/M phase. *TC *extract had similar effects in PG and WM451 cells (data not shown).

**Table 1:** Effect of *TC *extract on cell cycle distribution in PC-3M-1E8 cells.

*TC *extract concentration		Cell cycle (%)	
	
	G_0_/G_1_	S	G_2_/M
0 μg/ml	36.58 ± 1.39	12.49 ± 2.1	50.93 ± 0.93
1.67 μg/ml	23.61 ± 2.01 *	9.59 ± 0.53 *	66.79 ± 1.48 *
4.16 μg/ml	21.09 ± 2.64 *	10.74 ± 0.92 *	68.16 ± 1.86 *
8.33 μg/ml	6.88 ± 0.74 *	9.05 ± 3.33 *	84.07 ± 3.82 *
33.33 μg/ml	6.67 ± 1.54 *	9.71 ± 1.89 *	83.62 ± 3.38 *
66.67 μg/ml	9.46 ± 1.08 *	8.94 ± 1.08 *	81.60 ± 1.95 *

Cell cycle distributions in PC-3M-1E8 cells exposed to *TC *extract for 24 h, as determined by flow cytometry using propidium iodide staining. Values are mean ± SEM of 3 experiments. *Significantly different from 0 μg/ml (P < 0.05).

### Combination of 5-FU and *TC *extract produced synergistic effects on human cancer cells with lower cytotoxicity in normal cells than 5-FU alone

**Figure 5:**
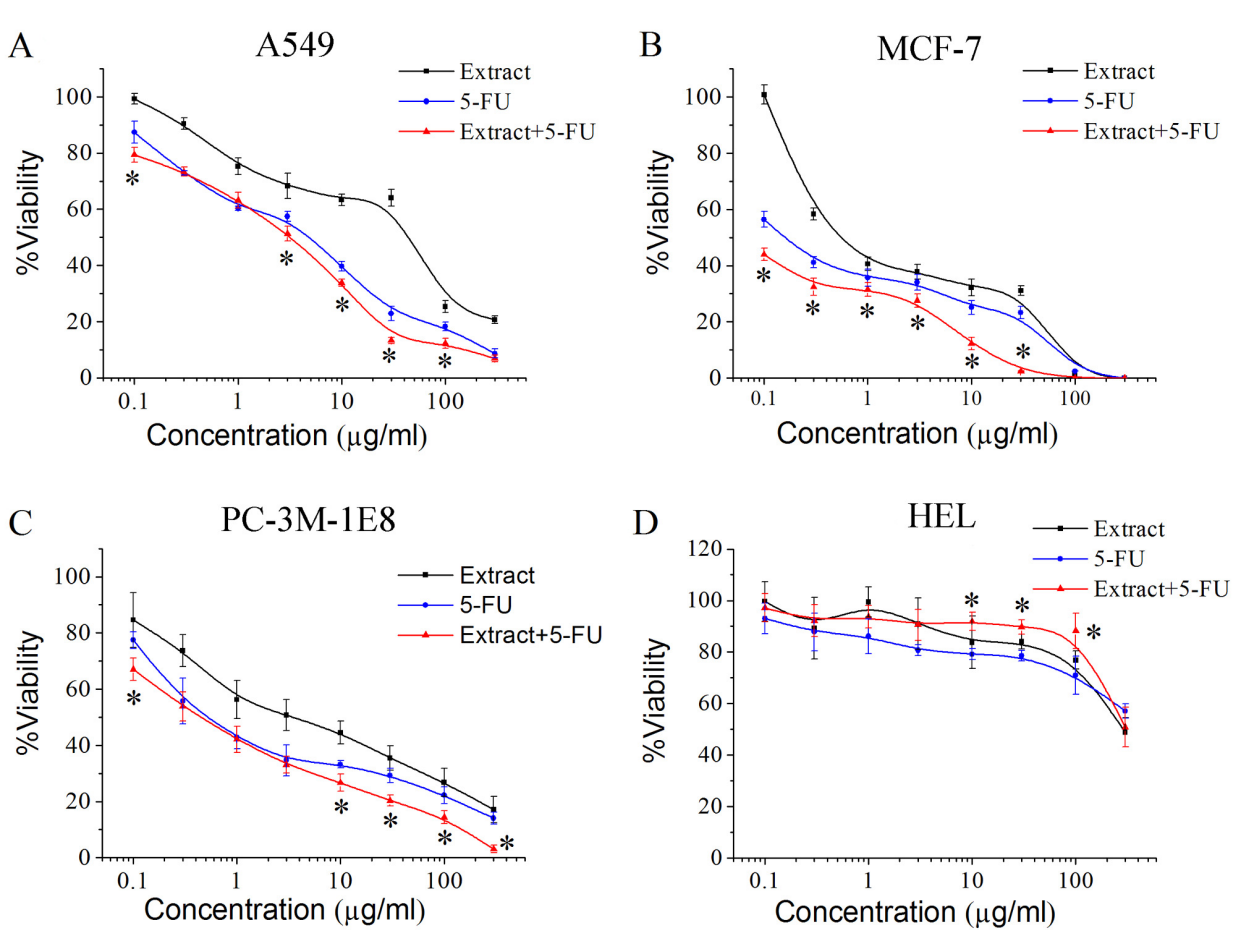
**Effect of *TC *extract and 5-FU on growth of human cancer cell lines and normal cells**. Human lung cancer cell line A549 (A), human breast cancer cell line MCF-7 (B), human prostate cancer cell line PC-3M-1E8 (C), and human embryo lung cell line HEL (D) were exposed to graded concentrations of *TC *extract (0.1-300 μg/ml), 5-FU (0.1-300 μg/ml), or the combination for 72 h. The proportion of live cells was quantified by ATP assay. Each point represents the mean ± S.D. (n = 5), and asterisks indicate significance at P < 0.05, compared with 5-FU alone.

In order to investigate the anticancer activity of a cocktail containing *TC *extract and 5-FU, cytotoxic studies were performed in cancerous (A549, PC-3M-1E8, and MCF-7) and normal (HEL) human cell lines. As shown in Figure 5, the viability levels of all cell lines decreased in a dose-dependent manner with increasing doses of different treatments. The survival of PC-3M-1E8 cells decreased from 84% to 17% with increasing doses (0.1-300 μg/ml) of *TC *extract, from 77% to 14% with increasing dose (0.1-300 μg/ml) of the 5-FU, and from 67% to 3% with the combination. In addition, *TC *extract concentrations above 10 μg/ml significantly enhanced the inhibitory effects of 5-FU at the same concentrations (Figure 5C). The same tendency was observed in A549 and MCF-7 cells, where the changes in cell survival levels with cocktail treatment were statistically significant compared with 5-FU treatment alone (Figure 5A and 5B). Furthermore, the combination of 5-FU and *TC *extract showed not only no significant inhibition of HEL cell growth compared with the individual treatments, even at a concentration of 100 μg/ml, but also lower cytotoxicity than 5-FU alone (Figure 5D).

**Figure 6:**
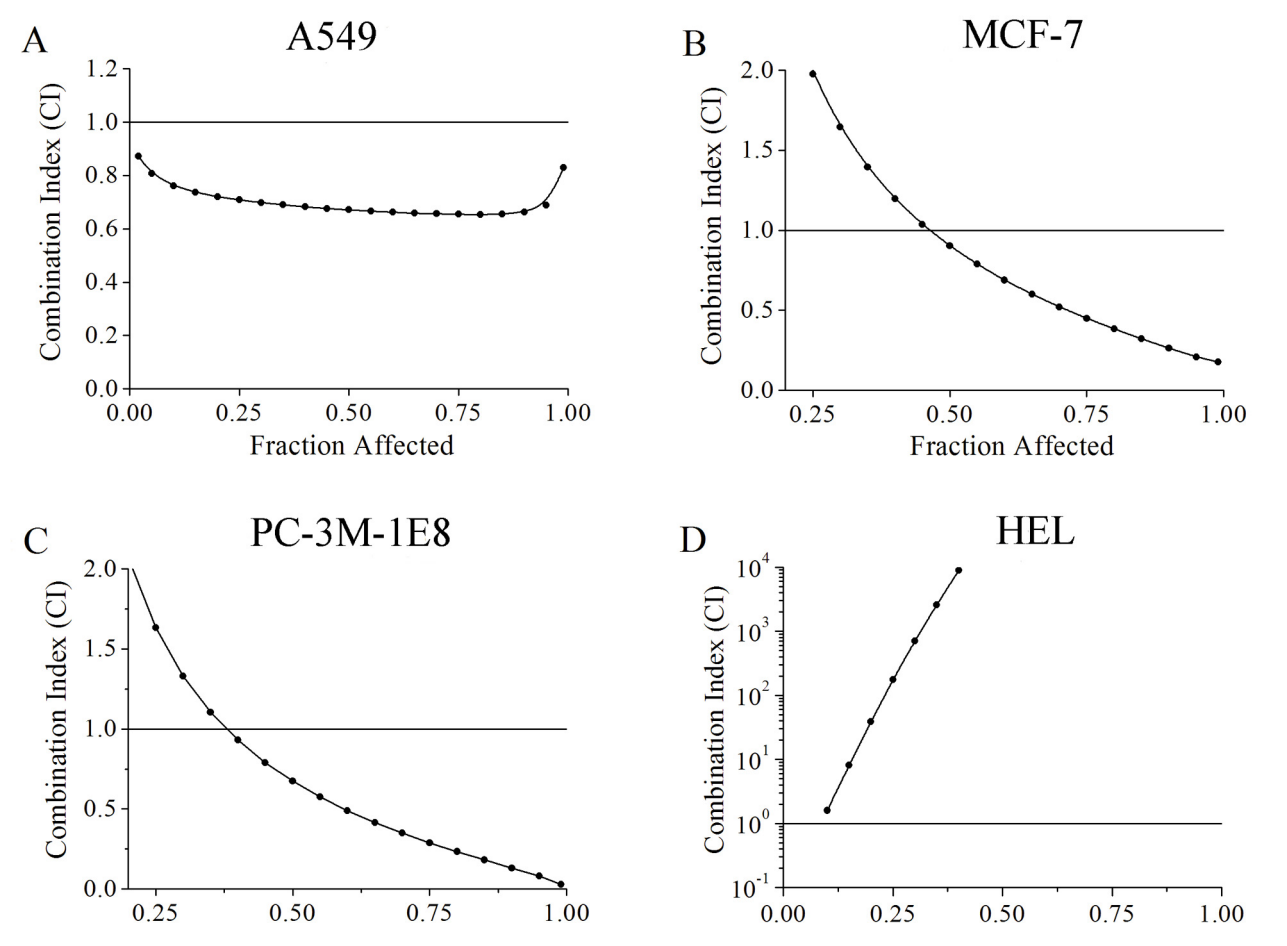
**Synergistic antitumor effect of the combination of *TC *extract and 5-FU *in vitro***. Combination index (CI) of *TC *extract with 5-FU in human lung cancer cell line A549 (A), human breast cancer cell line MCF-7 (B), human prostate cancer cell line PC-3M-1E8 (C), and human embryo lung cell line HEL (D) was calculated as described. Here, Fraction Affected on the x-axis denotes the proportion of cells affected (e.g., a Fraction Affected of 0.5 is equivalent to a 50% reduction in cell growth).

To determine whether the combined effects of the extract and 5-FU were synergistic, the CI value was calculated where CI < 1, = 1, and > 1 represent synergism, additive effect, and antagonism, respectively. The CI-fa curve shown in Figure 6 indicates that the 5-FU and the *TC *extract combination yielded synergistic effects with CI values ranging from 0.90 to 0.26 at different effect levels from IC50 to IC90 in MCF-7 cells (Figure 6B), with CI ranging from 0.93 to 0.13 for IC40 to IC90 in PC-3M-1E8 cells (Figure 6C), and with CI < 1 in A549 cells (Figure 6A). In contrast, the CI values were always > 1 in HEL cells (Figure 6D), indicating that the cytotoxic effect of the combination in normal cells was antagonistic. These results showed that the cytotoxic effect of the combination of *TC *extract and 5-FU was strongly synergistic in human cancer cells but antagonistic in normal cells.

**Figure 7:**
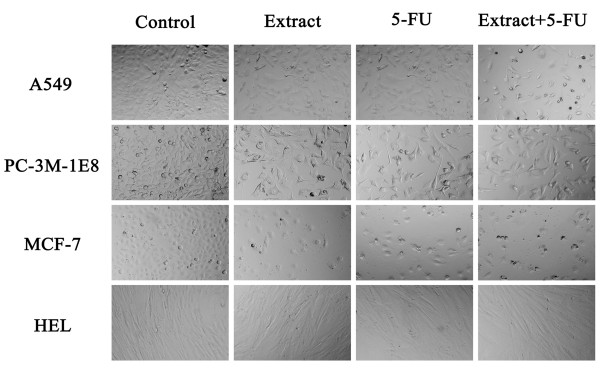
**Cytotoxicity in cancerous and normal human cell lines**. A549, PC-3M-1E8, MCF-7 and HELwere treated with 10 μg/ml *TC *extract, 5-FU or combination for 72 h. Cell images were captured using an Olympus IX51 inverted microscope (magnification × 100).

After treated with 5-FU, *TC *extract or combination for 72 h, cells images were captured. Each image was divided into nine equal pieces and one of them was chosen at random then the number of cells was counted. As shown in Figure 7, compared with the control, the cell numbers of the 3 human cancer cell lines decreased remarkably after treatment with either *TC *extract or 5-FU individually and even further after treatment with the combination. Nevertheless, the numbers of normal cells (HEL) were nearly the same, and the cells appeared healthy. The observations provided additional support to earlier results that the effect on inhibition of cell growth of combining *TC *extract and 5-FU was synergistic in human cancer cells and antagonistic in normal cells.

### *TC *extract does not affect the pharmacokinetics of 5-FU in rats

**Figure 8:**
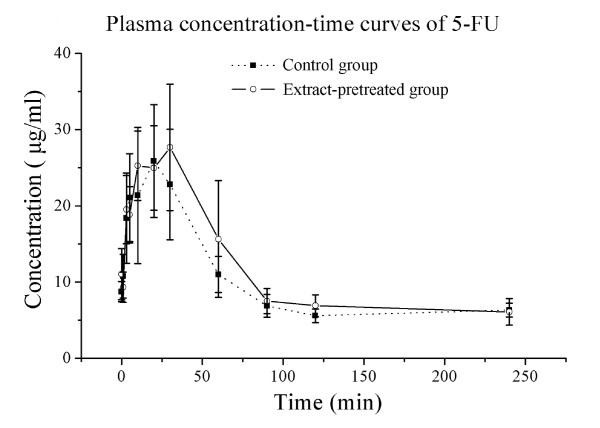
**Plasma concentration-time curves of 5-FU**. Blood samples were analyzed after a single i.p. administration of 48 mg/kg 5-FU to control rats (n = 5; black square) or rats pretreated with *TC *extract (1.5 ml/kg for 8 days, per os, n = 5; circle). Each value represents the mean ± SD of 5 rats.

The plasma concentration-time curves for the two groups after a single i.p. injection of 5-FU were adequately described by a first-order absorption two-compartment open model (Figure 8). The pharmacokinetic parameters were estimated using 3p87 software (Chinese Pharmacological Society, Beijing, China) and summarized in Table 2. None of the pharmacokinetics parameters, including the area under the curve (AUC), the time to reach the maximum concentration (T_max_), the maximum concentration (C_max_), or the absorption half-life (T_1/2_), showed any significant difference between the *TC *extract-pretreated group and the control group. These results indicates that *TC *extract did not affect the pharmacokinetics of 5-FU.

**Table 2:** Pharmacokinetic parameters of 5-FU (48 mg/kg) after a single i.p. injection to control rats (n = 5) or rats pretreated with *TC *extract (n = 5).

Parameter	Control group	Extract-pretreated group
AUC(μg/ml) × min	2317.81 ± 370.12	2864.17 ± 757.25
T_max_(min)	12.53 ± 4.88	16.23 ± 3.79
C_max_(μg/ml)	24.84 ± 7.45	23.32 ± 4.95
V/F(mg/kg)/(μg/ml)	1.77 ± 0.63	1.83 ± 0.41
T_1/2_(k_e_)(min)	58.5 ± 19.67	72.6 ± 8.93
T_1/2_(k_a_)(min)	3.05 ± 1.82	3.62 ± 1.14

AUC: area under the curve; T_max_: time to reach the maximum concentration; C_max_: maximum concentration; V/F: apparent volume of distribution; T_1/2_(k_e_): elimination half-life; T_1/2_(k_a_): absorption half-life. Values are means ± SD of 5 animals. There was no significant difference between the two groups.

## Discussion

In this study, we evaluated the anticancer activity of *TC *extract from the needles and twigs of *TC*, alone and in combination with 5-FU, *in vitro*. The pharmacokinetic interactions were further explored in rats. This study found that *TC *extract had a strong cytotoxicity to the 10 common human cancer cell lines (BGC-823, PG, WM451, Bel-7402, KB, HeLa, HL-60, MCF-7, A549, and PC-3M-1E8), which shown that it had broad-spectrum anticancer activity *in vitro*. Besides, it exhibited low toxicity to normal cells (mouse splenic T/B lymphocytes and HEL cells). *TC *extract inhibited cancer cell growth by inducing apoptosis and G_2_/M arrest. Moreover, the combination of *TC *extract with 5-FU showed higher inhibition in a number of human cancer cell lines (A549, MCF-7, and PC-3M-1E8) and lower cytotoxicity in normal cells (HEL). In addition, the pharmacokinetics of 5-FU were not affected by combination with *TC *extract. In summary, the effect of combining *TC *extract with 5-FU on cell growth inhibition was synergistic in cancer cells and antagonistic in normal cells. This cocktail may therefore have great pharmaceutical potential.

*TC *is one of the most extensively studied yew species, and more than 150 taxanes and other compounds have been reported [34]. Paclitaxel, one component of *TC*, is a popular anticancer drug used in clinical practice to treat ovarian, breast, and other carcinomas [23,31]. As an antimitotic agent, it induces cell apoptosis and G_2_/M arrest. In our study, HPLC fingerprinting identified 7 main taxoids in addition to paclitaxel in the extract. It has been reported that some of the taxoids found in the extract, such as baccatin III, 10-DAB, and cephalomannine, also interact with microtubules and inhibit the microtubule depolymerization process [14,23]. These could also inhibit human cancer cell growth as antimitotic agents. Therefore, the taxoids in *TC *extract may combine to kill human cancer cells by apoptosis and G_2_/M arrest.

Kano *et al. *[35] found that different sequences of paclitaxel and 5-FU administration had different efficacies and toxicities. For example, treatment with paclitaxel preceding 5-FU produced additive or synergistic cytotoxicity *in vitro*, while simultaneous exposure to paclitaxel and 5-FU showed mainly subadditive effects in A549, MCF-7, and WiDr cell lines. This is in contrast to our results, which demonstrated that the cocktail of *TC *and 5-FU had a synergistic anticancer effect in A549, MCF-7, and PC-3M-1E8 cells. One possible explanation for this discrepancy is different exposure times: in the experiment conducted by Kano *et al.*, the cell lines were exposed to paclitaxel and 5-FU for 24 h, while we treated for 72 h. The prolonged simultaneous administration of paclitaxel and 5-FU may restrain the antagonistic interaction [35]. The difference may also be due to the other taxoids besides paclitaxel found in *TC *extract. It has been reported that simultaneous treatment with docetaxel (an analog of paclitaxel) and 5-FU resulted in synergistic tumor inhibition in colon carcinoma xenografts in mice [36].

Interaction of herbals with drugs may also bring about changes in the pharmacodynamic and pharmacokinetic properties. Pharmacodynamic interactions may occur when a conventional drug has either synergistic or antagonist activity in relation to constituents of herbal products. Pharmacokinetic interactions are due to alteration of absorption, distribution, metabolism, or elimination of a conventional drug by an herbal product. The interaction may also increase/decrease the desired pharmacological effects of the drug [37,38]. For example, in the clinical trials, the effectiveness of haloperidol was enhanced when combined with *Ginkgo biloba *because of its antioxidant effects [37]. While some herbal products such as garlic and St John's Wort would decrease the effectiveness of a variety of prescription medications used to treat some cancers, AIDS, heart disease and organtransplant patients [38].

The side effects of anticancer agents are a serious problem in cancer chemotherapy and an effective anticancer approach with potent activity and minimal side effects is highly desirable [15]. It is well known that some of the side effects of 5-FU are gastrointestinal such as nausea, vomiting, and myelosuppression. The association between toxicity and high 5-FU plasma levels has been reported since the late 1970s [39-41]. Studies have shown that marked elevation and prolongation of 5-FU levels in the plasma would increase toxicity of 5-FU [29,40,41]. Pharmacokinetic data can be used to predict the likelihood of an interaction between the *TC *extract and 5-FU. Our results indicated that *TC *extract did not affect the pharmacokinetics of 5-FU in rats. Furthermore, at the cellular level, the cocktail had lower cytotoxicity in normal cells despite a synergistic anticancer effect. Therefore, as a cocktail, combination of 5-FU with *TC *extract may show a possibility for enhancing the efficacy. Still, the exact mechanisms of the effects need to be further researched in our study, such as drug-metabolizing enzymes and drug transporter systems involved.

Lung cancer is the most common cause of cancer-related death in men and women worldwide. Breast cancer is the most prevalent cancer among women and affects approximately one million women. Prostate cancer is one of the most prevalent types of cancer in men. To expand future possiblity of the cocktail used in clinic, we chose these common and prevalent cancer cell lines for the study of combined treatment. And we found that the cytotoxic effect of the combined *TC *extract with 5-FU was strongly synergistic in the three cancer cell lines (lung cancer - A549, breast cancer-MCF-7, and prostate cancer - PC-3M-1E8). Other advantages of *TC *extract are that *TC *needles and twigs can be obtained from artificial cuttage, making the extract constantly available, easy to prepare and inexpensive. In contrast, the extremely small quantity of paclitaxel in *TC *[31,42] makes purified paclitaxel rather expensive, which limits its use, particularly in developing countries.

## Conclusions

In this study, we reported broad-spectrum anticancer activity of the extract of *TC *needles and twigs produced by artificial cuttage. This is the first study to explore a cocktail of *TC *extract and 5-FU, which had synergistic effects on human cancer cells and low cytotoxicity in normal cells. These results provided important information for clinical cancer therapy. Additional, preclinical and clinical development is certainly needed to explore the uses of the *TC *extract-5-FU cocktail for cancer therapy, and the exact mechanism of the synergistic effect should be further investigated.

## Abbreviations

5-FU: 5-fluorouracil; AUC: area under the curve; CI: combination index; C_max_: maximum concentration; fa: fraction affected; PI: propidium iodide; T_1/2_(k_a_): absorption half-life; T_1/2_(k_e_): elimination half-life; T_max_: time to reach the maximum concentration; V/F: apparent volume of distribution.

## Competing interests

The authors declare that they have no competing interests.

## Authors' contributions

WHS carried out synergistic effect experiments, required added experiment and drafted the original manuscript. JPQ designed and carried out the pharmacokinetic experiments and drafted the original manuscript. CXG carried out the pharmacokinetic experiments and helped draft the original manuscript. WY carried out extract identification experiments. JLD performed the statistical analysis. WW carried out anticancer activities experiments. MLZ carried out the pharmacokinetic experiments. WDL made substantial contributions to conception. MH designed the study, performed mechanistic experiments and coordination and finalized the manuscript. All authors read and approved the final manuscript.

## Pre-publication history

The pre-publication history for this paper can be accessed here:

http://www.biomedcentral.com/1472-6882/11/123/prepub

## Supplementary Material

Additional file 1 **Supplementary figure S1. Chemical structures of the 8 main peaks**. Peaks: 1, taxine A (5.00%); 2, 10-DAB III (1.45%); 3, 9-hydroxyl-13-acetylbaccatin III (9.38%); 4, 7-xyl-10-DAT (9.63%); 5, baccatin III (8.32%); 6, 10-DAT (6.31%); 7, cephalomannine (2.67%); 8, paclitaxel (2.25%). Click here for file
