# Supplementary material for: Anticancer activity of an extract from needles and twigs of Taxus cuspidata and its synergistic effect as a cocktail with 5-fluorouracil
Source: BMC Complement Altern Med. 2011 Dec 2;11:123. doi: 10.1186/1472-6882-11-123 (PMC3260134; doi:10.1186/1472-6882-11-123)
Supplement: Additional file 1 — Supplementary figure S1. Chemical structures of the 8 main peaks. Peaks: 1, taxine A (5.00%); 2, 10-DAB III (1.45%); 3, 9-hydroxyl-13-acetylbaccatin III (9.38%); 4, 7-xyl-10-DAT (9.63%); 5, baccatin III (8.32%); 6, 10-DAT (6.31%); 7, cephalomannine (2.67%); 8, paclitaxel (2.25%). [file 1472-6882-11-123-S1.DOC]

**Additional File**

**Anticancer activity of an extract from needles and twigs of *Taxus cuspidata* and its synergistic effect as a cocktail with 5-fluorouracil**

**Supplementary Figure**

**Supplementary Figure S1.**

**Chemical structures of the 8 main peaks.** Peaks: 1, taxine A (5.00%); 2, 10-DAB III (1.45%); 3, 9-hydroxyl-13-acetylbaccatin III (9.38%); 4, 7-xyl-10-DAT (9.63%); 5, baccatin III (8.32%); 6, 10-DAT (6.31%); 7, cephalomannine (2.67%); 8, paclitaxel (2.25%).

**Supplementary Figure S1.**

**
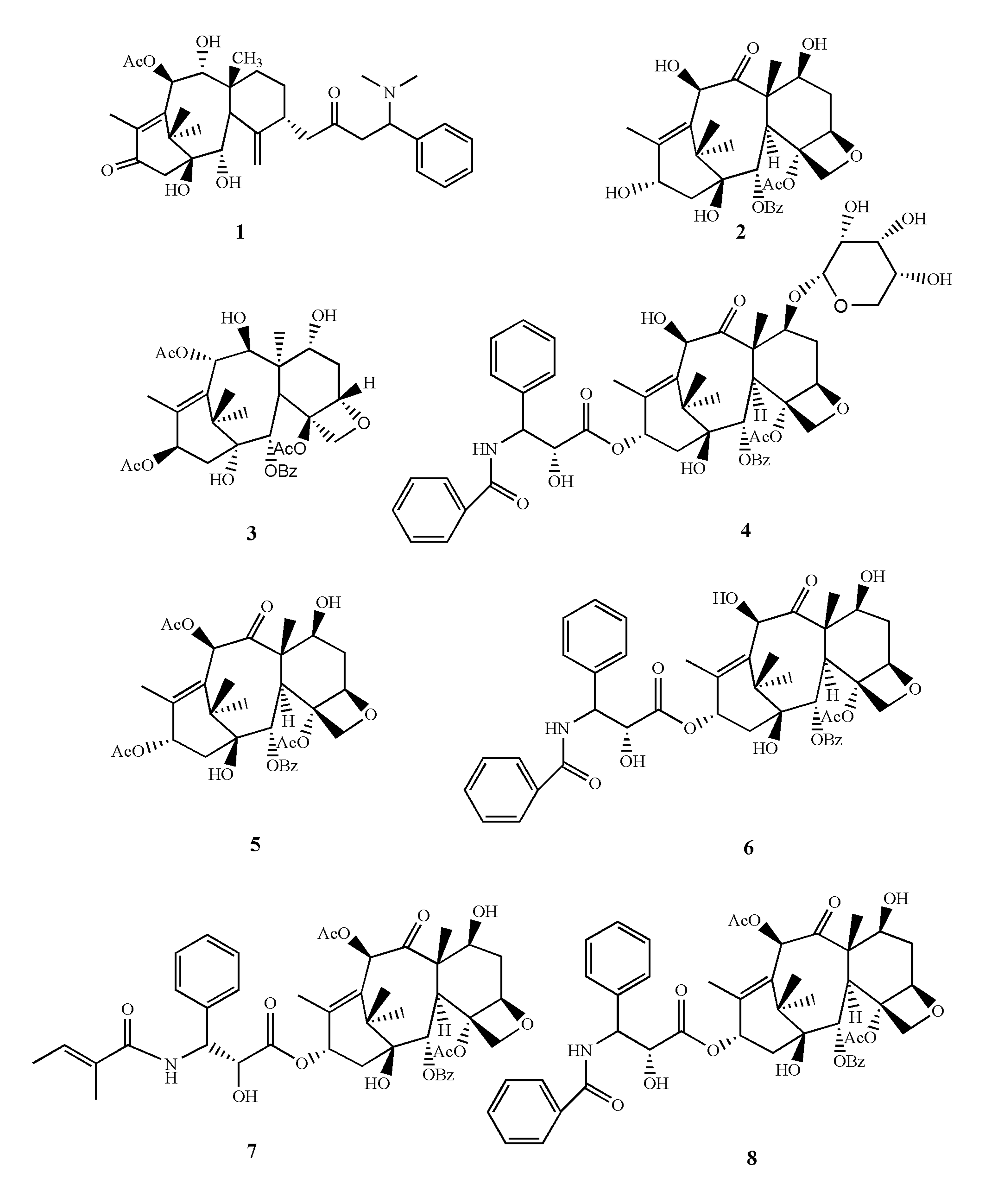
**
